# Optimal Energy Management System of IoT-Enabled Large Building Considering Electric Vehicle Scheduling, Distributed Resources, and Demand Response Schemes

**DOI:** 10.3390/s22197448

**Published:** 2022-09-30

**Authors:** Liu Fei, Muhammad Shahzad, Fazal Abbas, Hafiz Abdul Muqeet, Muhammad Majid Hussain, Li Bin

**Affiliations:** 1 School of Electric and Information Engineering, Tianjin University, Tianjin 300072, China; 2 Department of Electrical Engineering, Muhammad Nawaz Sharif University of Engineering and Technology, Multan 60000, Punjab, Pakistan; 3 Department of Electrical Engineering Technology, Punjab Tianjin University of Technology, Lahore 54770, Punjab, Pakistan; 4 Electrical, Electronic & Computer Engineering, School of Engineering and Physical Sciences Heriot-Watt University, Edinburgh EH14 4AS, UK; 5 School of Electrical and Electronics Engineering, North China Electric Power University, Beijing 100096, China

**Keywords:** demand response, electric vehicle, energy storage system, energy management system, microgrids, smart grid

## Abstract

In the energy system, various sources are used to fulfill the energy demand of large buildings. The energy management of large-scale buildings is very important. The proposed system comprises solar PVs, energy storage systems, and electric vehicles. Demand response (DR) schemes are considered in various studies, but the analysis of the impact of dynamic DR on operational cost has been ignored. So, in this paper, renewable energy resources and storages are integrated considering the demand response strategies such as real-time pricing (RTP), critical peak pricing (CPP), and time of use (ToU). The proposed system is mapped in a linear model and simulated in MATLAB using linear programming (LP). Different case studies are investigated considering the dynamic demand response schemes. Among different schemes, results based on real-time pricing (58% saving) show more saving as compared to the CPP and ToU. The obtained results reduced the operational cost and greenhouse gas (GHG) emissions, which shows the efficacy of the model.

## 1. Introduction

**Figure 1:**
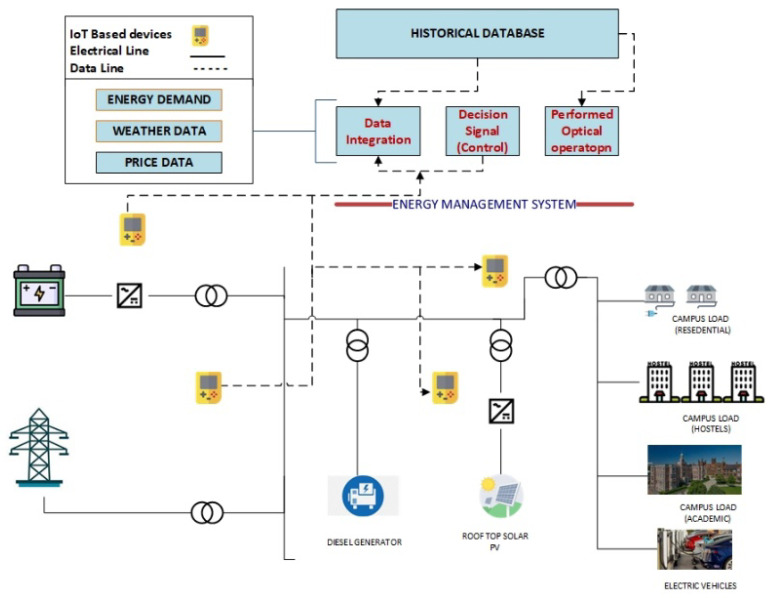
General system architecture.

Energy is a need in every field of life. As the population is increasing, energy demand is also increasing. In the conventional energy system, the energy is produced only from fossil fuels [1,2,3]. As fossil fuels are being depleted and are also the cause of carbon emission, environmentally friendly energy generation is needed [4,5,6,7]. In a smart power system, the microgrid plays a very important role due to the integration of renewable energy resources. Microgrids can be worked in grid-connected and off-grid-based scenarios. Grid-connected large-sized buildings are also a very effective load in the power system network [8,9,10,11,12]. Smart grids [13] evolved as a remedy for many difficulties inside the current electrical system as a result of a worldwide surge in electricity needs. The use of sustainable energy foundations, power storing, and enabling the user to make some decisions about energy usage are all innovative components of smart grids [14,15,16]. Microgrids are key components of smart grids, and because large buildings are major consumers of energy, their usage may be efficiently managed in conjunction with the assistance of an energy management system [17,18,19,20,21]. The Internet of Things (IoT) is a complex embedded system that uses software and cloud systems connected to the internet [22]. The IoT is very useful for integrating various technologies with microgrids. It addresses different devices with smart microgrids such as diversified technologies. It also interacts with a cyber system that is necessary for the physical system [23]. The main benefit of the IoT is that it enables the end-user to be more interactive and responsive as compared to the other technologies [24]. Additionally, the IoT enables various sensors, actuators, and other storage and protective components. So, IoT is very beneficial for smart grid operators and utility companies. IoT can also be applied in industries, healthcare centers, and smart cities. The data received from the various devices are collected and interpreted for further analysis. The end-user energy consumption behavior can be obtained from the IoT-based devices that can be used for load forecasting. The general microgrid system architecture is shown in Figure 1.

The electrical grid, in particular, must be intelligent, dependable, and flexible in order to cope with increased peak demand and avoid dim-outs and shutdowns. Solar and wind energy are being used to prevent the installation of big centralized generators. Smart grids were formulated to improve a variety of grid issues as well as to make them more intelligent and flexible [25]. The rapid growth of sensing, automation, and information and communications technology (ICT) covered the technique for the production of cost-effective smart grid solutions. A smart grid was created to upgrade the current electrical grid by employing information and communication technology to collect and use data in an automated manner to produce and distribute electricity more effectively and reliably [26]. The term “smart grid” is frequently used to refer to the technology for electric vehicles (EVs). EVs emit no greenhouse gases, drive quietly, and have considerably better fuel efficiency than internal combustion engine vehicles [27]. They are critical for the world’s long-term transportation. Certainly, several businesses and colleges have chosen to replace their business fleets using environmentally friendly automobiles. Smart microgrids are much more efficient, reliable, and cost-effective when electric vehicles are used as storage and renewable energies are used as distributed generators [28]. It is important to construct a smart microgrid depending on the requirements of the users, selecting the kind and scale of the most economic and premium technologies available, including renewable power plants and high-efficiency cogeneration units, as well as electrical and thermal storage systems [29]. One of the major challenges in the smart grid field is the energy management system (EMS) for end-users. The smart grid contains the interconnection of microgrids, and thus energy exchange between them has the potential to lower microgrid operation costs and decrease the amount of load-shedding required by utilizing various programs such as demand response. Furthermore, the advantages of a microgrid include distributed generation (DG) with the energy storage system, a dependable system that maximizes the profit of active customers by competing in the energy trading market.

In the next sections, the literature review is presented in Section 2, while the system description is given in Section 3. Section 4 presents the mathematical modeling, while the results and discussion are presented in Section 5. The main findings are summarized in the conclusion in Section 6. 

## 2. Literature Review

In the existing system, microgrid energy management systems are studied using different techniques. The main purpose of the demand response is to change the energy consumption behavior of the end-user by offering some incentives. Some studies are reviewed here to explore the research gap in this era. Reference [30] introduces the EMS for implementation of PV generation and an ESS on an existing campus. To reduce energy consumption and operate the battery optimally, a simulated annealing algorithm was used. Wind and photovoltaic (PV) energy have acquired great popularity around the world as a way to avoid using fossil fuels and cut carbon emissions. Due to lower solar system installation and operation costs, the high penetration of small and large-scale PV applications has increased significantly; generation, on the other hand, is inconsistent and heavily dependent on climate conditions, resulting in power swings during inclement weather. In [31], the authors propose an ideal microgrid system based on locally accessible energy resources for a real physical location with real-time power demand. The HOMER PRO software set is used for extensive technical and financial research using a multi-decade growth method to establish the ideal energy system, which has not been seen before in the literature. The findings of the extensive research show that photovoltaics generate the majority of the electricity produced in all situations. In off-grid systems, the renewable proportion is higher, and significant improvements such as 92% to 100% were observed, while the previous range was 63% to 80%.

In [32], the intended task includes intelligence collection and compilation, a programming model, economic evaluation, installation, and power running of diesel generators, as well as the creation of a recommended microgrid. The results from the proposed framework showed that a 1.8 MW generator-based microgrid can operate autonomously in a variety of situations, with load distribution, load and sag changes, genset interruption, and islanding grid modes. A DER-based system is a multi-input and multi-output system that consists of smart technologies [33]. To overcome the power prices or the conservation influence of distributed energy resource systems, everyday process arrangement is critical. The optimal featuring of the DER system is found by solving a stochastic multi-objective linear programming problem to minimize the predicted power prices and CO_2_ emissions. Buildings, according to Farid Farmani and reports from the US Department of Energy, play a critical role in the energy demand industry. The majority of electricity consumption in the United States is attributable to buildings [34]. 

One of the most advanced technologies is combined cooling, heating, and power systems. They meet all thermal, cooling, and electrical requirements [35]. The goal of the essay by Seyed Mehdi Hakimi is to define the size and location of multiple elements in microgrids, including renewable energy resources (RERs) [36]. Different parameters including reliability, wind speed unpredictability, solar irradiation, load, and load increase are taken into account [37]. Numerous limits are based on consideration of voltage, frequency, resources, and the capacity of the energy storage system. The methodology is validated on a wind turbine/PV/FC/hydrogen tank microgrid system, and the best sizing is determined [38]. The authors of [39] create a BESS numerical model that can be used to analyze power grid applications. Two test groups might be created for the proposed laboratory technique. The first is to acquire the SOC curve centered on the system’s OCV (SOC-OCV curve) as well as the battery’s capability curve. It comprises complete charge and full emission cycles with constant power (DoD = 100%). The SOC-OCV curve must be estimated to define SOC since it provides a reliable reference term. A dynamic battery energy storage system (BESS)-based energy consumption cost model presented in [40] analyzes the dispatch cost as a function of the dispatch power. 

The proposed model also considered the relationship between the voltage of the storage, its current, and the SOC, which is equal to the connection between conversion efficiency and power and SOC. Initial simulations show that utilizing the suggested approach for microgrid operation optimization instead of the LCOE reduces microgrid operation costs by up to 12%. In [41], a sliding mode observer capable of monitoring SOC while considering capacity factors is proposed by the author. The observer’s task is to change capacity depending on the decaying factor. The root mean square error of the measured and estimated voltage is 0.013 V, whereas the modeled voltage is 0.12 V. The mean difference between observed and modeled capacity is 0.14 Ah. PV systems with capacities ranging from 40 to 60 kW were used, and the PV Watts Calculator created by the National Renewable Energy Laboratory was used to calculate their solar power generation profiles for a typical day. 

In [42], the author proposes a day-ahead battery energy scheduling strategy that takes into account the costs of battery degradation caused by charging and discharging cycles. The Rainflow algorithm, particle swarm optimization, and scenario techniques are all incorporated into a framework for solving the function. Simulation findings suggest that the proposed strategy is successful and can save up to 40% on operational costs. Authors in [43] decreased the running cost of an isolated microgrid by using economic scheduling to determine the ideal battery capacity. As a result, the cost of real-time battery operation is estimated by taking into account the depth of discharge at each time interval. Due to a lack of battery storage, the microgrid is vulnerable to load shedding, resulting in high operating costs and instability, according to modeling data. Reference [26] describes a comprehensive toolset for resolving power quality problems in a microgrid by synchronizing the working periods of the microgrid’s generating resources and loads. By merging a MILP-based power development procedure with a reiterative energy eminence enhancement method, this work provides an integrated tool for effectively mitigating power quality challenges in a microgrid operation. The EMS seeks to attain the cheapest overall system price by synchronizing the deployment periods of the microgrid’s DERs and loads. In [44], the authors present research on the HEMS to provide a full evaluation of history and evaluate several DR programs and smart technologies with load scheduling controllers. The authors of [45] demonstrate several settings on a real-world MG system.

In addition, extensive simulations on a variety of typical test systems are performed to compare the estimation difficulty of the projected approach towards the standard of ADMM and to assess the technique’s scalability. The study of bi-level optimal operation planning considers microgrids and battery storage systems as two separate users with opposing agendas. For the topmost optimal control problem, the overall cost of the microgrid was minimized, which included the price of trading energy across MGOs and BSSs, the expenditure of exchanging power among DSOs and MGOs, and costs connected with MTs, PVs, and loads. The authors of [46] constructed two optimization methods for scheduling the microgrid operation in grid-connected and islanded modes. To ensure optimal operating in each mode, the proposed technique may shed loads, define photovoltaic generating levels, and control the charging or discharging level of the energy storage system. Two MILP-based optimization problems using predicted data as inputs are used in the proposed method. The findings suggest that the recommended schedule is successful in optimally operating the microgrid.

According to [47], the market for electric vehicles (EVs) is growing with the move toward more distributed, clean, and renewable energy sources. The use of electric vehicles switches a significant portion of mobility energy demand to structure electricity meters. As a result, approaches for integrating energy efficiency into the building and transportation sectors are becoming more significant. Three different case scenarios were analyzed to find the maximum number of electric vehicles that the LaCER building transformer can support. The true load data gathered for the building are divided into discrete time intervals for a transformer loss of life (LOL) calculation. The main goal of the proposed study in [48] is to create a microgrid that is connected to the grid with solar generation and storage batteries to meet campus demand load while lowering grid reliance. The microgrid was modeled and simulated using the Hybrid Optimization Model for Electrical Renewables (HOMER) program. According to HOMER’s techno-economic analysis, the campus microgrid has two viable possibilities. The most efficient PV systems are grid-connected PV systems with battery storage, followed by grid-connected PV systems deprived of battery storage. In [49], the authors proposed a novel system for the USC campus microgrid, in which three key components of the system are presented in real time: semantic information integration, machine-learned demand forecasting models, and load curtailment detection using CEP. The new approach, for the simulation results, best calculates the MG schedule by utilizing the entire capacity of various MG facilities, such as CHP units, WTs, electricity storage, and heat storage. The authors of [50] introduced a multi-microgrid-based system considering the combined cooling and heating load and system power for cooperative as well as optimal energy management. Each BMG can meet its energy demands by trading excess power and heat energy with the utility grid and EEN. The recommended technique reduced daily thermal energy losses by 84.36% over the whole network during the summer period. The network’s daily heat losses were reduced by 28.74% throughout the winter season. 

In [51], the authors assessed the importance of distribution system reliability in a microgrid system. Concerning the cost of energy trading, the main supply, the cost of microgrid-based energy production, and the load curtailment expenses are all included in the microgrid operation cost. In a microgrid, the cost of battery operation is believed to be insignificant. Microgrid interruptions could result in a drop in revenue of up to USD 80 per kWh. The annual operating cost is USD 140,497 per year. HRDS installation would cut the annual operating cost to USD 126,644. By executing demand response and reaping the benefits of daily market price fluctuations, the storage will reduce its yearly operation cost to USD 119,236 per year. In [52], the authors proposed a real-time digital simulator (RTDS) used to analyze the distribution system in real time and investigate system dynamics. The response of the battery deployed in the microgrid is shown in the simulation for islanded operation. The model was also useful in determining the impact of a PV system and its continuously varying generation on the load bus voltage. 

In [53], the authors proposed an EMS for the ideal functioning of the Savona Campus smart polygeneration microgrid (SPM), with the goal of lowering total production costs while meeting all thermal and electric network restrictions. The optimal dispatching of low voltage microgrids has been calculated and reported using an efficient method. The entire design allows for the representation of both the behavior of dynamic devices such as storage batteries and the restrictions of the electric network in great detail.

From the above discussion, it is deduced that the optimal scheduling for a large-scale building considering the demand response program has been ignored. By considering the demand response schemes, a comprehensive analysis is carried out to investigate the effect of various DR schemes on the operational cost of the system. So main contributions of this paper are as follows:

- Optimal scheduling of the proposed system considering the various types of demand response.
- EV-based charging and discharging modeling is considered for the real-time analysis of smart buildings in developing countries.
- The effect of different demand responses on the operational cost of the building is analyzed.
- In the next section, the system description is presented considering the main objective of the proposed model.

## 3. System Description

An institution on a college campus in Pakistan was employed in this research. The main campus of the institution spans 388.5 hectares (960 acres). There are currently 51 colleges, departments, and institutes in the system. The campus for investigation is 28.3 hectares (70 acres). There are five departments on the campus, as well as a library, hostel, and accommodation for employees. The principal source of electricity is Multan Electric Power Firm, a provincial distribution company (MEPCO). This article suggests combining a PV scheme, a BESS, and EVs using the transmission classification to attain vigor stability and minimize power usage costs. The proposed systems consider the demand response program. 

### 3.1. Demand Response Type

Some details of the DR are time of use (ToU), critical peak pricing, and real-time pricing. All these are described here. 

#### 3.1.1. Time of Use (ToU) Pricing

The ToU depends on the time of energy demand and its consumption. The time interval is very important and is divided into the peak and off-peak hours [54]. 

#### 3.1.2. Critical Peak Pricing (CPP)

This DR scheme is rarely used during the year. It is similar to the ToU but has some differences in the interval of time during peak hours [21]. 

#### 3.1.3. Real-Time Pricing (RTP)

In this type of pricing, the electricity tariffs typically change hourly, reflecting the fluctuations in the price of the wholesale electricity market. Typically, the consumers are notified on a deadheaded hour-ahead basis [2]. RTP is becoming very popular in DR programs and smart homes. 

### 3.2. Photovoltaic System

In terms of solar energy usage, Pakistan is one of the world’s luckiest countries. On the horizontal position, average annual sunlight hours range from 1700 to 2200, and usual lunar radioactivity roughly has the value of 2000 kWh/m/year. Similarly, the daily power is 200–250 Watts/m/day, and the yearly output observed is 2400 kWh/m^2^/year on the angle of 30° slant facing south [40,41]. The design of a 600 kW PV structure on the building’s rooftops, as well as a solar parasol at a charging station for EVs, has been suggested.

### 3.3. Energy Storage System (ESS)

The importance of energy storage is very high as it provides the backup for the load shedding or peak hour times. When the stored energy is needed, it can be converted back to electrical energy [43]. Along with renewable energy sources, the ESS is becoming an important part of smart grid and microgrid architecture. An ESS not only helps to solve the intermittent nature of renewables but also allows for peak shaving and demand response strategies to be implemented [44]. ESSs also help grid operators power system networks and manage energy storage [45]. BESS [46,47,48,49] energy storage with flywheels [50], supercapacitors [51], storing compressed air energy [52], and storing hydrogen energy [53] are several different varieties of ESSs. Lithium-ion batteries have grown widely popular as BESS batteries due to their low self-absorption proportion, good consistency, high influence and vigor efficiency, and long lifespan [54]. In this research, a lithium-ion battery-based system with a rating of 100 kWh storage space was selected. This battery size was selected to hold excess power generated by the PV system while also providing electricity during power disruptions or topmost eras.

### 3.4. Electric Vehicles (EVs)

Many researchers are working on the evolution of traditional transportation systems into autonomous mobility on demand (AMoD) systems [55,56] due to rising environmental problems. Autonomous vehicles are projected to revolutionize urban environments, while electric vehicles are now helping to decarbonize the transportation industry. Electric vehicles (EVs) are a prominent feature of the current era, contributing significantly to the greening of road transportation. Furthermore, because of the high amount of power necessary to charge their batteries, a big number of electric vehicles pose a threat to power systems and additional difficulties due to the reliability and cost concerns. 

**Figure 2:**
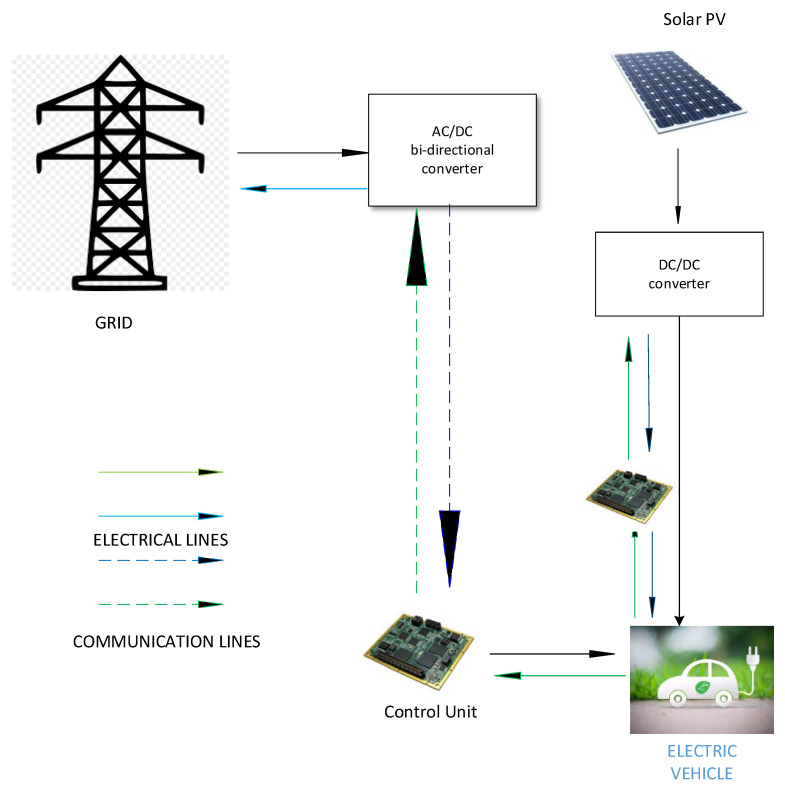
Proposed system architecture.

In this scenario, the use of vehicle-to-grid (V2G)-based technology can resolve these problems. As in V2G technology, electric vehicles serve as storage devices, storing energy during off-peak hours and assisting the grid during peak hours. Teachers and students, during office hours, can store their cars at the charging station, from 9:00 to 3:00, to evaluate the influence of introducing EVs to the planned system. PV generation and grid power will be used to charge electric vehicles. Even while the automobiles are parked, they can charge and discharge. EVs will aim to reach their full charging capacity. When there is a grid outage or a large increase in load demand, EVs will function as a source to supply electricity to the system. Figure 2 shows how electric vehicles will be integrated into the system [57].

## 4. Mathematical Modeling

The mathematical modeling of the various components is described in the following subsections. 

### 4.1. PV Modeling

The output supremacy of PV modules is mostly determined by solar irradiation and temperature and can be estimated as follows:

(1) $$\mathrm{Ppv}=N\eta inv\;[Pn,r\frac{Gk}{Gk},r\left(1+r\left(Tc-Tr\right)\right]$$

Electric vehicles (EVs) here are the transformer productivity, whereas *P* represents the solar PV output power and n shows the total number of components in the PV system. In Equation (1), *G* represents sunlight at time instant *t*, *G* represents solar insolation during source test conditions, *T* represents the temperature of the cell, and *T* is the temperature gradient for the reference test conditions.

(2) Tc=Tamb+9No−20/0.8)Gk

The surface temperature is *T*, while the nominal operating cell temperature is N. The method beneath can be used to compute the installed PV capacity, *P* [58].

(3) Pc=ηpv.A.ωp

where at the peak solar insulation, *A* is the solar panel area and is the PV efficiency. PV running expenses are regarded as a constant cost of repairs throughout time. Through the use of an inverter, the PV system’s DC power output is transformed into AC electricity. The following expression is used to calculate the inverter rating using the power *P* of the solar modules and the typical efficiency of the inverter.

(4) $$Ci\eta v=PA\frac{100}{\eta inv}$$

The data on the sunlight used here come from [59].

### 4.2. Energy Storage Modeling 

The storage system has some binary variables as expressed in Equation (5).

(5) $$X_{Ess}^{c}\cdot X_{ESS}^{d}\in \left\{0,1\right\}$$

The equations below show the behavior of the charging as well as discharging power limitations. They illustrate that if the charging power is $X_{Ess}^{c}$ = 0, the charging level will also be zero. If it is in the not charging mode, it is also not in the discharging mode $X_{ESS}^{d}$ = 0.

(6) $$P_{min}^{c}\leq P_{t}^{c}\leq X_{ESS}^{c}\;P_{max}^{c}$$

(7) $$P_{min}^{d}\leq P_{t}^{d}\leq X_{ESS}^{d}\;P_{max}^{d}$$

where the charging and discharging powers are *P* and *P*, respectively. The battery must be in a charging or draining condition at any one time.

(8) $$X_{Ess}^{c}+X_{ESS}^{d}\leq$$

If the battery is entirely drained, the system may be destroyed. As a consequence, *ESS* charging and discharging must stay within the parameters of the models below [60].

(9) $$\frac{SC\left(t\right)-SC^{max}}{100}C^{ESS}\;\leq P^{ESS},t$$

(10) $$\frac{SC\left(t-1\right)-SC^{min}}{100}C^{ESS}\;\geq P^{ESS},t$$

where *SC* denotes the battery’s state of charge and *P* denotes the battery’s power output. The battery’s *SC* level at time instant t is resolute through the preceding state and intended via the following equivalence:

(11) $$SC\left(t\right)=SC\left(t-1\right)-\frac{P^{ESS},\;t\;\times \;100}{C^{ESS}}$$

The following equation must be satisfied to prevent the battery from aging too quickly:

(12) $$SC^{min}\leq SC\left(t\right)\leq SC^{max}$$

Regardless of how frequently the ESS is utilized, the *SC* level at the beginning and conclusion of each optimum phase will be the same.

(13) $$SC\left(to\right)=SCt^{end}$$

### 4.3. EV Constraints

If an electric vehicle is linked to a power outlet, it can be used as a storage system. Using the method below, the *SC* for the kth *EV SC*, which would be the transition time between entry and exit, can be computed [58].

(14) $$SC\left(k,t\right)=SC\left(k,t-1\right)+[\eta_{EV}^{c}\;P_{k,t}^{c}-\frac{P_{k,t}^{d}}{\eta_{EV}^{d}}$$

The following equation can be used to compute the demand for electricity of the kth *EV* at time *t*, *P* [59].

(15) $$P\left(k,t\right)=P_{k}S_{T}\omega_{t}h_{t}$$

If *P* stands for the kth EV’s rated power, s stands for the EV’s connection state at time *t*, w stands for weekdays, and h stands for working time, then by combining the power demand of each EV, the aggregated EV power consumption for several EVs may be computed.

(16) $$\begin{array}{ccc} P^{EV}\left(t\right)=\left\{\overset{N}{\underset{K=1}{\sum }}P\left(k,t\right)\right. & & \left(\mathrm{time}\;\mathrm{to}\;\mathrm{change}\;\mathrm{EVs}\right)\\ 0 & \mathrm{else} & \end{array}$$

(17) $$P_{k,min}^{c}\leq P_{k,t}^{c}\leq P_{k,max}^{c}$$

(18) $$P_{k,min}^{d}\leq P_{k,t}^{d}\leq P_{k,max}^{d}$$

The state of the charge (SC) for the kth hour of EV at time t should be within limitations, and the storage device’s overall health must be within limits, to avoid condition impact.

(19) $$SC^{min}\left(k,t\right)\leq SC\left(k,t\right)\leq SC^{max}\left(k,t\right)$$

### 4.4. Grid Connection

The microgrid connection has access to the full power exchange limit, which enables the energy owned from the grid or supplied to the grid to stay below the restrictions shown below [60].

(20) $$-P_{min}^{g}\leq P_{g}\leq P_{max}^{g}$$

The following equation has to be fulfilled in order to achieve supply–demand equilibrium:

(21) $$P\left(g,t\right)+P\left(pv,t\right)+P\left(ESS,t\right)+P\left(EV,t\right)=P\left(load,t\right)$$

#### 4.4.1. Objective Function

The aim is to reduce the price of everyday electricity costs by grouping sources of power efficiently, as shown below, which is described as a linear optimization problem.

(22) $$\min k=\overset{T}{\underset{t=1}{\sum }}⬚\left[K^{g\;}\left(t\right)+K^{PV}+K^{ESS}\left(t\right)+K^{EV}\left(t\right)\right]$$

where

(23) $$K^{g}\left(t\right)=\left(P^{g}\left(t\right)\right)\;R^{g}\left(t\right)$$

(24) $$K^{PV}\left(t\right)=\left(P^{PV}\left(t\right)\right)R^{PV}\left(t\right)$$

(25) $$K^{ESS}=\left(P^{ESS}\left(t\right)\right)R^{ESS}\left(t\right)$$

(26) $$K^{EV}=\left(P^{EV}\left(t\right)\right)R^{EV}\left(t\right)$$

In the above Equations (22)–(26), *K* is the energy cost, while other costs such as $K^{g}\left(t\right)$, $K^{PV}\left(t\right),\;K^{ESS}\left(t\right),\;and\;K^{EV}\left(t\right)$ are various costs: electricity costs for the grid, photovoltaic system, *ESS* cost, and *EV*, respectively. Similarly, the unit price for any time is expressed by (*t*), which is given in the above equation.

#### 4.4.2. Solution Methodology

Robust linear programming is used to tackle the optimization problem. It is a method for determining the best solution for a given situation that takes into account linear combinations. It uses less computing time to reach the optimal solutions and is rarely employed at campus microgrid locations. The following is a general definition of linear programming:

(27) fxmintx

such that

(28) $$\left\{\begin{array}{c} A.x\leq b,\\ A_{eq}.x=b_{eq,}\\ ub\leq x\leq lb. \end{array}\right.$$

In the above Equations (27) and (28), some terms such as *f*, *x*, *b*, *beq*, *lb*, and *ub* express the vectors, whereas *A* and *A_eq_* represent the matrices. In MATLAB, the objective function is solved using the interior point method. For use of monitoring and control, the devices are based on IoT [61,62,63,64,65].

## 5. Results and Discussion

**Figure 3:**
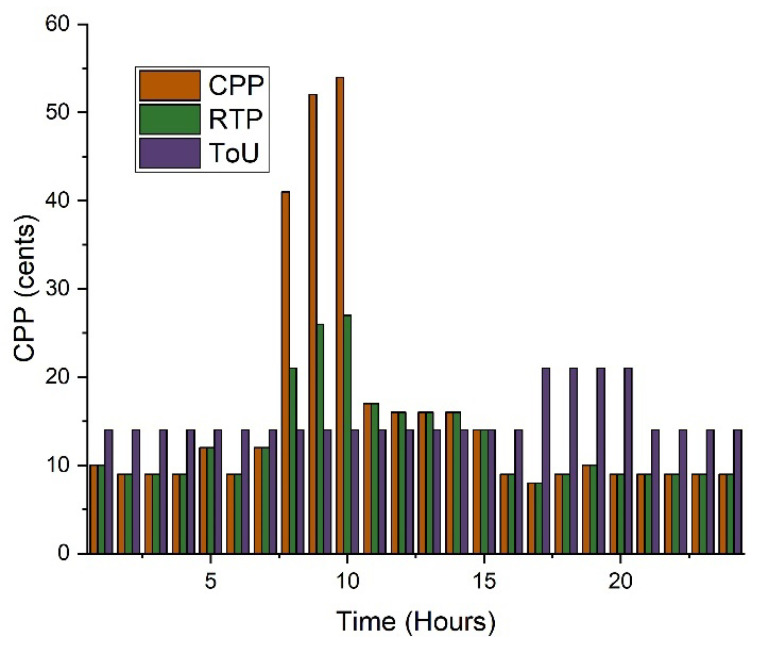
Three pricing schemes.

To investigate the best result, the following cases are analyzed using linear programming in MATLAB. These scenarios vary in terms of the components and characteristics they employ [66,67,68,69,70]. Microgrid analysis and planning require connecting the cases to a reference or base case. The reference scenario is the current state of the system prior to microgrid deployment. This paper explains the reference case as well as the other cases that are related to it. Figure 3 shows the pricing schemes of the demand response. 

**Figure 4:**
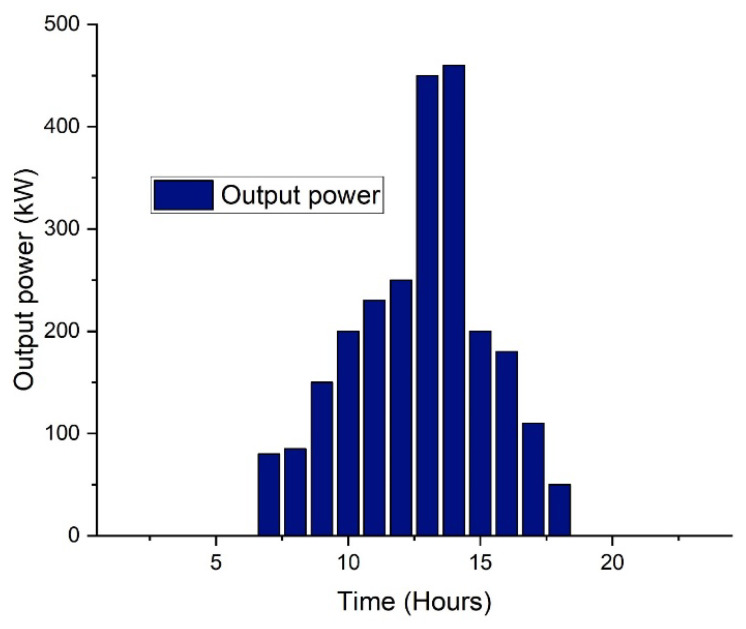
Solar PV output power patter.

**Figure 5:**
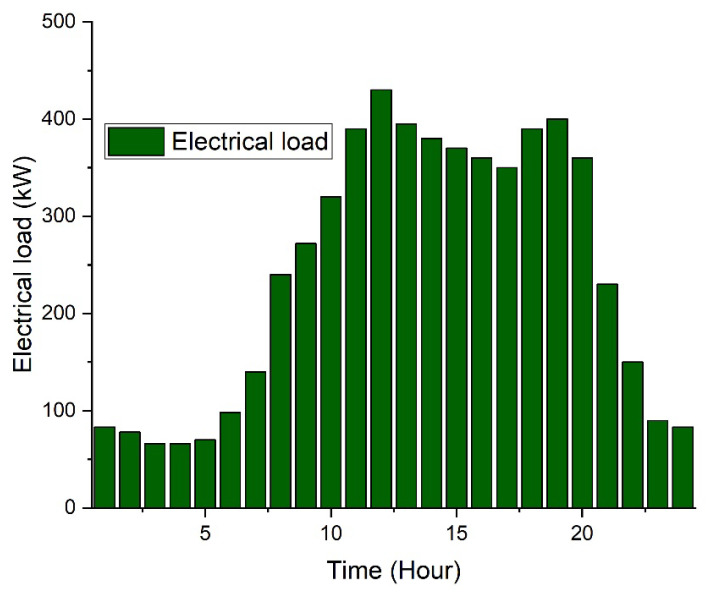
Building energy demand pattern.

The impacts of photovoltaic and energy storage systems such as both static and mobile adoption, as well as the grid, are evaluated. Figure 4 shows that from night until 7:00 a.m., there is little or no sunshine to generate electricity [71,72,73]. The PV module then uses sunshine to generate electricity, reaching its peak around midday. Figure 5 depicts the college’s daily average load profile, which is normal in the nonappearance of any generation or storage in a microgrid.

As illustrated in Figure 5, the average academic, dormitory, and residential loads make up the predicted day load profile. The load outline shows a surge in petitions throughout institution hours due to educational load and late at night time due to household load.

Figure 5 expresses the building energy daily consumption pattern, while Table 1 expresses the case studies that will be presented in the next sections. 

The details of all mentioned cases are presented in the next subsections. 

### 5.1. Case 01, Analysis Based on Real-Time Pricing 

In the demand response, real-time pricing (RTP) is considered to check the effect on the operational cost.

*i.*  *Without Scheduling*

**Figure 6:**
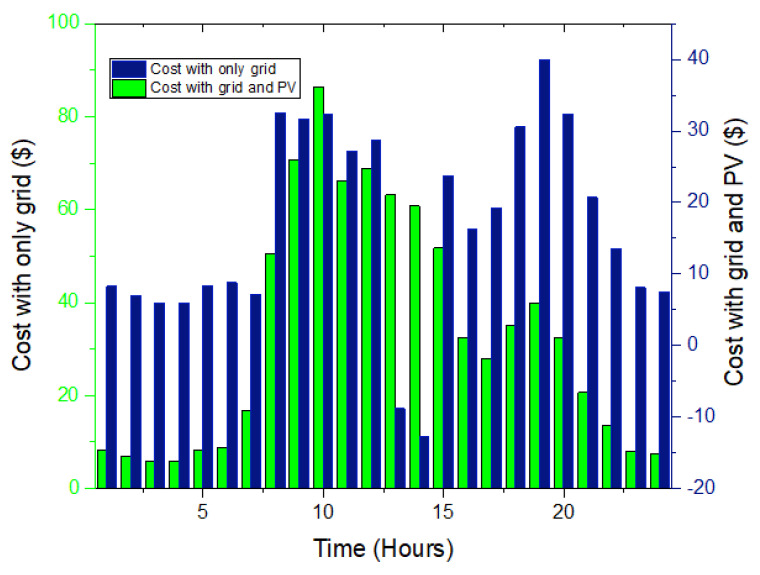
Cost behavior without and with solar PV.

The load profile changes dramatically after installing the PV system with ESS. This is because the difference between the original load profile and PV production determines the final load profile when a PV system is combined with some load. This is plainly demonstrated in Figure 6, where there has been a reduction in the load by a significant amount, which corresponds to the PV system’s production.

In this situation, cases with and without solar PV are taken into account. The utility grid is the only source of power if solar PV is not considered. In this scenario, the cost of power using RTP is estimated to be USD 797.31 and USD 394 per day, respectively, as shown in Table 2. These situations are used as a base case for further comparisons.

The effect of solar PV is significant in that it reduces the energy cost by 55% as compared to the grid-only case. In the next case, scheduling-based analysis is carried out considering the mobile and stationary energy storage systems.

*ii.*  *With Proposed Scheduling*

**Figure 7:**
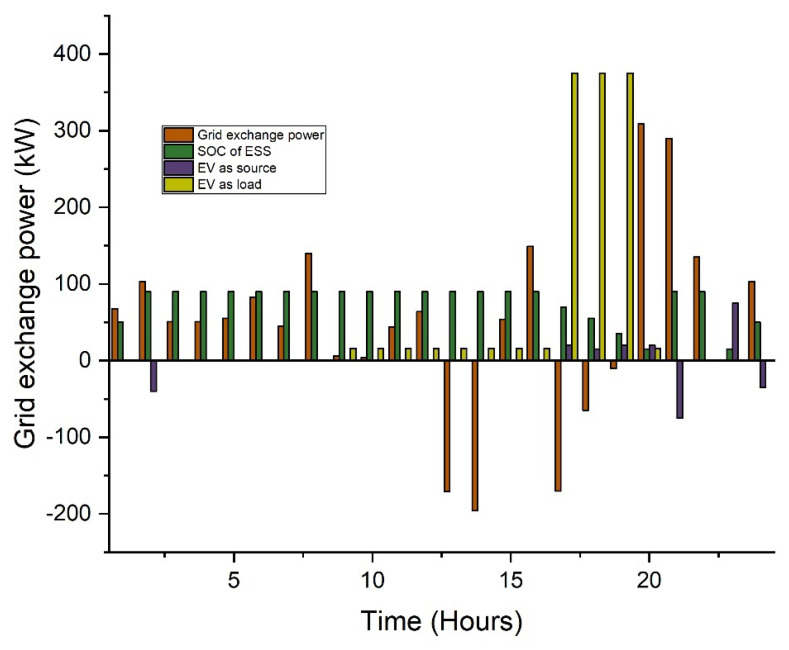
Scheduling based analysis in the RTP scheme.

In this case, two types of scheduling are analyzed: without incentive and with proposed incentivized-based scheduling. The proposed incentivized system offers the end consumer about a 10% cost reduction in the utility cost. In this way, the consumer feels comfort as compared to conventional schemes. The system overload and congestion issues may resolve using the proposed scheduling. Additionally, the effect of incorporating electric vehicles and battery energy storage is also considered. For the case study, a total of 100 EVs are considered. The average usage distribution with EVs included in the system is analyzed. EVs will aim to reach their full charging capability. While functioning as a load, EVs can store energy and then send it to the grid during peak times. The overall electricity price for this scenario is USD 354 per day, according to simulation data as shown in Table 2. Figure 7 shows that from 09:00 to 15:00, less electricity is exported to the grid since the majority of the power is demanded to charge the electric vehicles. 

Similarly, when the incentivized analysis is carried out, the cost is further reduced, as shown in Table 2. This is the optimal scenario as compared to all previous results and can be implemented in the real-time system in the campus microgrids. 

### 5.2. Case 02, Analysis Based on Critical Peak Pricing 

In this case, the CPP is analyzed using the above-mentioned details.

*i.*  *Without Scheduling*

**Figure 8:**
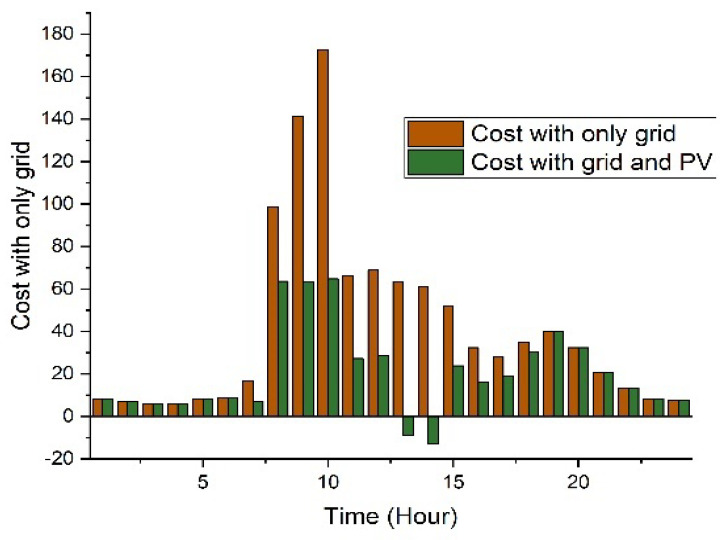
Cost analysis without and with solar PV.

In this situation, no PV, ESS, or EVs are taken into account. The utility grid is the only source of power. In this scenario, the cost of power that uses the CPP pricing is estimated to be USD 1002 and USD 489 per day without and with solar PV, respectively. Figure 8 shows the cost pattern of this case.

*ii.*  *With Proposed Scheduling*

**Figure 9:**
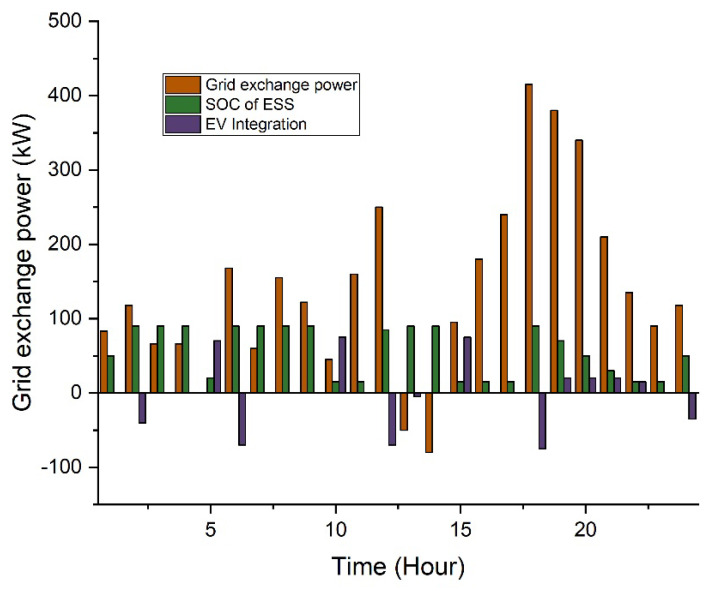
Scheduling-based analysis in CPP.

Electric vehicles serve as a basis of electricity in this situation, supplying loads and the grid with stored energy. The EVs are expected to perform as a 100 kWh storage system. The overall electricity price for this example is USD 480 and USD 451 per day for the conventional scheduling and incentivized scheduling, respectively, according to simulation data. Table 3 shows the results of the CPP, while the Figure 9 shows the results of this case. 

### 5.3. Case 03, Analysis Based on Time of Use Pricing

The analysis based on time of use is presented to differentiate the various demand response techniques.

*i.*  *Without Scheduling*

**Figure 10:**
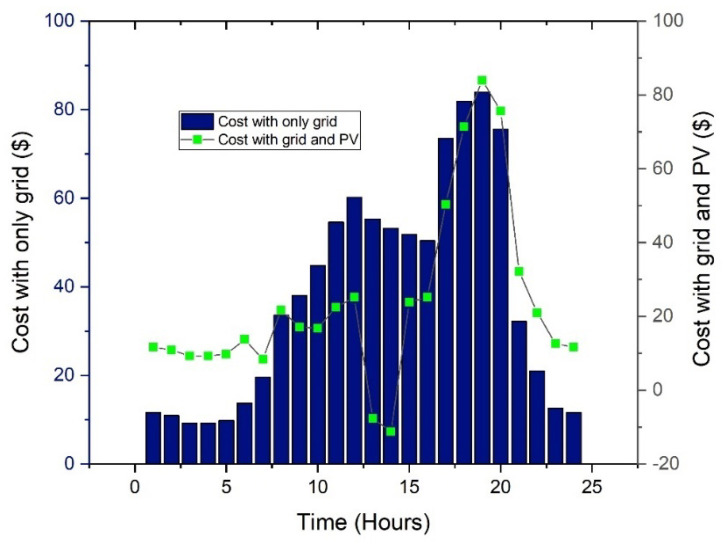
Cost analysis grid and solar PV.

In this situation, no PV, ESS, or EVs are taken into account. The utility grid is the only source of power. In this case, the cost of power that uses the ToU pricing is estimated to be USD 918 per day. Similarly, after the solar PV is considered, the cost is reduced by USD 565 as shown in Figure 10 and Table 4. 

In the next subsection, scheduling-based analysis is carried out to investigate the behavior of the different parameters.

*ii.*  *With Proposed Scheduling*

In this case, two types of scheduling are analyzed: without incentive and with proposed incentivized-based scheduling. The proposed incentivized system offers the end consumer about a 10% cost reduction in the utility cost. In this way, the consumer feels comfort as compared to the conventional schemes. The system overload and congestion issues may be resolved using the proposed scheduling. Additionally, the effect of incorporating electric vehicles and battery energy storage is also considered. For the case study, a total of 100 EVs are considered. 

**Figure 11:**
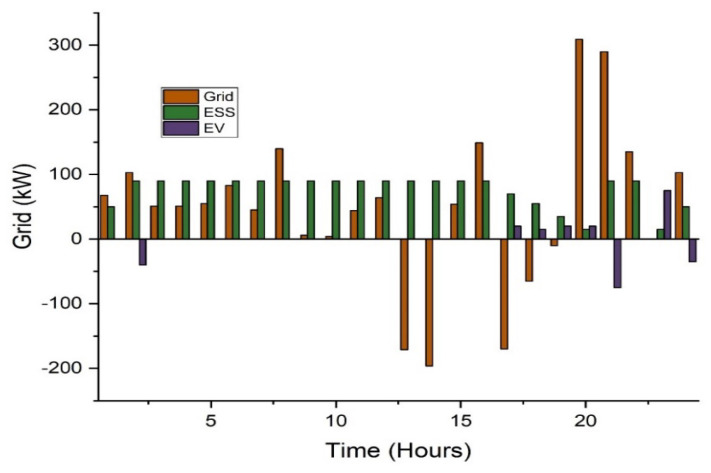
Scheduling-based analysis in ToU.

The average usage distribution with EVs included in the system is analyzed. EVs will aim to reach their full charging capability. While functioning as a load, EVs can store energy and then send it to the grid during peak times. The overall electricity price for this scenario is USD 354 per day, according to simulation data as shown in Table 4. Figure 11 shows that from 09:00 to 15:00, less electricity is exported to the grid since the majority of the power is demanded to charge the electric vehicles. 

In the proposed scheduling, the cost is reduced to USD 498 from USD 526. There is a significant reduction when using the ToU-based system. 

In the foregoing instances, the integration of DERs with the current grid and the best scheduling of available energy resources are investigated. Among the three cases, the real-time pricing is better than the other scenarios. In the Table 5 the comparison of the costs are presented. 

## 6. Conclusions

In this work, an IoT-enabled microgrid is scheduled to minimize the operational cost of large buildings. The system comprises renewable and non-renewable energy resources, an ESS, and EVs. The proposed model is mapped in linear programming and simulated in MATLAB. Various case studies are carried out considering the price-based demand response schemes. In the demand response schemes, incentives based on real-time pricing, critical peak pricing, and time of use are analyzed. The cost savings were analyzed and found to be 45%, 55%, and 58% in ToU, CPP, and RTP, respectively. Furthermore, the proposed scheduling strategy was also investigated and compared with the conventional demand response schemes, revealing significant results. The analysis revealed that real-time pricing is very cost-economical for both the utility and the end-users. The cost was reduced by 58%, which is much better than the already existing works. In the future, the stochastic modeling of all sources and demand will be addressed to carry out real-time analysis.

## Figures and Tables

**Table 1:** Case studies and different proposed scenarios.

Scenarios	Grid Availability	Solar PV	BESS and EV
Case (01) RTP Case (02) CPP Case (03) ToU	✓	-	-
	✓	✓	-
	✓	✓	✓
Proposed Scheduling (Incentivized 10%)	✓	✓	✓

**Table 2:** Results of case study RTP scenarios.

Scenarios		Cost Grid Availability (USD)	Cost Solar PV (USD)	BESS and EV	Saving (%)
Case (01) RTP	i.	797.31	-	-	-
	ii.	394		-	50
	iii.	354			55
Proposed Scheduling (Incentivized 10%)		717	355	301	58

**Table 3:** Results of case study CPP scenarios.

Scenarios		Cost Grid Availability (USD)	Cost Solar PV (USD)	BESS and EV	Saving (%)
Case (02) CPP	i.	1002	-	-	-
	ii.	489		-	51
	iii.	480			52
Proposed Scheduling (Incentivized 10%)		902	440	451	55

**Table 4:** Results of case study ToU scenarios.

Scenarios		Cost Grid Availability (USD)	Cost Solar PV (USD)	BESS and EV	Saving (%)
**Case (03) ToU**	i.	918.54	-	-	-
	ii.	565		-	38
	iii.	526			42
Proposed Scheduling (Incentivized 10%)		826	508	498	45

**Table 5:** Comparison of existing and proposed costs.

Ref.	Year	Application	Technique	Remarks	Savings
[74]	2018	Campus µG	MILP	Peak demand	5.32%
[75]	2018	Residential Level	MILP	Frequency-based regulation	7%
[76]	2019	Residential µG	LP	Grid for the mode of outage	16%
[77]	2020	Campus µG	MILP	Peak mitigation	23%
[78]	2021	Campus µG	MILP	ESS degradation cost, peak demand	5.27%
Proposed Model	2022	Campus µG	LP	ESS, Demand response, EV	58%

## Data Availability

Not applicable.
